# 9/11 Health Update

**DOI:** 10.3390/ijerph18126383

**Published:** 2021-06-12

**Authors:** James E. Cone, Albeliz Santiago-Colón, Roberto Lucchini

**Affiliations:** 1 World Trade Center Health Registry, Division of Epidemiology, New York City Department of Health and Mental Hygiene, New York, NY 10013, USA; 2 World Trade Center Health Program, National Institute for Occupational Safety and Health, Cincinnati, OH 45226, USA; yvx8@cdc.gov; 3 School of Public Health and Social Work, Florida International University, Miami, FL 33199, USA; rlucchin@fiu.edu

## 1. Introduction

This Special Issue of the *International Journal of Environmental Research and Public Health* is dedicated to increasing the scientific information available about the long-term effects of exposure to the 2001 World Trade Center disaster. We address emerging health problems, chronic diseases including cancer and mortality, as well as research methods and intervention strategies. The following are summaries of the sixteen articles included in this issue.

## 2. WTCHP First Decade of Research

The James Zadroga 9/11 Health and Compensation Act of 2010 was signed into law on January 2011 and the WTC Health Program (WTCHP) was established to provide medical monitoring and treatment of covered health conditions for responders and survivors of the 9/11 attacks. The WTCHP also maintains a research program aimed to inform the clinical care of program members. In their review, Santiago-Colón et al. [1] describe the research findings of the first decade of the Program and discuss future research directions. The focus of research has been characterizing the burden and etiology of WTC-related health conditions. Future research needs point to translational research that enhances the care of this population.

## 3. Cognition

There is growing evidence suggesting that 9/11 exposures may increase the risk of mild cognitive impairment (MCI) occurrence. In their article, Daniels et al. [2] provide an overview of the scientific workshop convened by the WTCHP on cognitive aging and impairment in the 9/11 exposed population. The workshop centered on evaluating the evidence of causal associations between 9/11 exposure, PTSD, and the risk of adverse cognitive function; the potential health burden from mild cognitive impairment (MCI); the research gaps in this area; and the steps needed to manage MCI risk and improve care. As a next step, the WTCHP is pursuing a research agenda targeting a broad range of studies on cognitive decline and other issues related to aging in the 9/11-exposed population.

To evaluate how mental health comorbidity is associated with cognitive impairment, Alper et al. [3] investigated the incidence of confusion or memory loss (CML) and its association with the number of mental health conditions among WTC Registry enrollees aged 35–64 at wave 4 survey. CML was reported among 20% of enrollees. Results showed a dose–response relationship between CML and the number of mental health conditions ranging from one to three.

In a follow-up study, Singh et al. [4] examined the interrelationships between PTSD (a well-established risk factor for cognitive dysfunction), depressive symptoms, and environmental exposure that may also be related to cognitive decline among FDNY firefighters and EMS providers. Results showed that PTSD symptoms completely mediated the association between WTC exposure level and self-reported cognitive decline while depressive symptoms largely mediated this association. These findings suggest that WTC-exposed populations could potentially benefit from interventions targeting PTSD and depression to mitigate cognitive decline.

## 4. Posttraumatic Growth

Pollari et al. [5] have studied the effects of direct 9/11 exposure on posttraumatic growth, the concept developed by Tedeschi and Calhoun that, following a trauma, a person may experience positive psychological growth. PTG was measured using the Posttraumatic Growth Inventory (PTGI). They found that higher 9/11 exposure, higher scores on the Posttraumatic Check List, high social integration, more social support and higher self-efficacy were associated with increase in PTGI score. Future studies are needed to identify and test interventions using modifiable factors that may increase PTG.

## 5. Food Intake Restriction

Kwon et al. [6] proposed an intervention among overweight WTC-exposed New York City firefighters involving the low-calorie Mediterranean diet facilitated by technological means. Outcomes to be measured include lung function, measures of vascular injury and quality of life. If implemented successfully, this would be a good example of the use of surveillance data to guide public health interventions.

## 6. Mortality among New York City Fire Department Workers

Colbeth et al. [7] studied the mortality among Fire Department of New York City rescue and recovery workers. The researchers used NIOSH’s Life Table Analysis System to generate Standardized Mortality Ratios with the US general population as the comparison group. The researchers also examined the association between WTC exposures and mortality using Cox proportional hazards regression. Not surprisingly, given the impact of the healthy worker effect, all-cause mortality was much lower than the comparison population. Several specific causes including deaths from mesothelioma and building fires were elevated but not statistically significantly so. Unlike studies of other WTC-exposed cohorts, no apparent association of mortality was noted with WTC exposure.

## 7. Record Linkage

Asher et al. [8] reviewed the history of record linkage with a focus on methods pertinent for WTC Registries. The researchers identified the current models for probabilistic data linkage, outlined the steps required, and potential for bias. These methods will likely become more important over time as the WTC-exposed population ages, and new data sources become available.

## 8. Intentional Self-Medication with Alcohol

Garrey et al. [9] studied the intentional self-medication of 9/11-related PTSD symptoms with alcohol (ISMA) among World Trade Center Health Registry (WTCHR) enrollees. They found that ISMA is relatively common in the WTC-exposed population, with an observed relationship between severity of PTSD and frequency of ISMA. Binge drinking was reported more frequently with increased numbers of PTSD clusters among those with ISMA. Overall, this study supported the self-medication hypothesis.

## 9. Somatic Symptoms in WTC-Exposed

Alper et al. [10] studied somatic symptoms that have been reported by enrollees in the WTCHR. The researchers evaluated the association between severity of WTC injuries and non-specific psychological distress with somatic symptoms. The pattern of somatic symptoms was compared with results from a nationally representative German study. They found that both physical and mental health conditions contribute to the development of somatic symptoms. The number of enrollees reporting a very high symptoms level was significantly increased compared with the German population sample. The implications of this study may be the significant resources that may be devoted to treatment of these patients, a lesson for the World Trade Center Health Program.

## 10. Translational Impact of WTC-Disaster Research

Madrigano et al. [11] evaluated the potential public health impact of WTC disaster-related research on PTSD and cancer using the National Institute for Environmental Health Sciences’ Translational Framework. This review identified several areas in need of additional studies, including effectiveness of current PTSD treatments, further research on cancer screening practices and cancer therapy options relevant to WTC-exposed populations. The NIEHS Translational Framework is complex and conceptually challenging, but was used successfully to identify future research opportunities to address two important disease outcomes.

## 11. Cancer

An elevated risk for multiple cancers has been shown for the WTC affected community members residing in South Manhattan on 9/11 (also called WTC “Survivors”). Dr. Shao et al. [12] describe a new REDCap-based pan-cancer platform developed at the WTC Environmental Health Center (EHC) Data Center hosted by the New York University. This database includes pathology reports and biomarker data of 3440 confirmed cancer cases reviewed by a cancer epidemiologist, a pathologist, physicians, and biostatisticians. Cases were diagnosed after 11 September 2001 until 31 December 2019, and obtained from WTC EHC clinical and pathological reports, and New York State cancer registries.

A comprehensive description of 2561 cancer cases detected at the EHC was provided by Durmus et al. [13] A total of 2999 cancer diagnoses in these subjects included 2534 solid tumors (84.5%) and 465 lymphoid and hematopoietic tissue cancers (15.5%), with forty-one different cancer types. Of not is a high rate of rare cancers, including male breast cancers and mesotheliomas have been identified among the WTC survivors.

Characteristics of lung cancer among survivors, in particular, were examined by Durmus et al. and coauthors [14]. Although lung cancer incidence has been consistently found to be lower in the WTC-exposed population compared with the general population, it is of particular interest due to the nature of the WTC-disaster exposure involving many lung irritants and carcinogens. No generalizations to the wider population of WTC-exposed are possible due to the self-referred nature of this cohort, but nevertheless the analysis of the differences between types of cancers found among men and women, and smokers versus non-smokers suggests opportunities for future studies in this population.

## 12. Lung Disease

Another study on the WTC Survivors examined the persistence of lower respiratory symptoms (LRS) of uncontrolled asthma affecting individuals residing in lower Manhattan with previous WTC exposure to airborne particles. Reibman et al. [15] reported that out of 60 patients seen at the EHC, who were uncontrolled at baseline, only 11 participants (18%) gained controlled status as defined by the Asthma Control Test after a three-month run-in period on high-dose inhaled corticosteroid and long-acting bronchodilator. After the treatment, uncontrolled subjects showed higher rates of upper airway symptoms, whereas controlled subjects showed persistent bronchial hyper-reactivity (BHR) and upper airway hyper-reactivity as measured by paradoxical vocal fold movement (PVFM). Persistent upper and lower airway hyper-reactivity may respond to standard asthma treatment, whereas persistent LRS necessitate additional diagnostic evaluation on the upper airway.

A two-year longitudinal assessment was conducted on 8530 WTC-exposed firefighters with lung injury by Putman et al. [16] A total of 1629/8530 (19.1%) firefighters initiated inhaled corticosteroid/long-acting beta-agonist ICS/LABA treatment conducted for >2 years. Forced Expiratory Volume in 1 s (FEV_1_), wheeze, and dyspnea were independently associated with prolonged ICS/LABA treatment. This study shows that high risk for treatment was identifiable from routine monitoring exam results years before treatment initiation.

## 13. Conclusions

The World Trade Center disaster of 2001 continues to impact exposed populations in diverse ways. This is increasingly documented in the scientific literature, as demonstrated in this update issue as we approach the disaster’s 20-year anniversary. We need to focus on interventions to reduce preventable morbidity and mortality in these populations, and future research is needed on topics pointed to in this Issue.
